# Anti-Inflammatory Activities of Compounds Isolated from the Rhizome of *Anemarrhena asphodeloides*

**DOI:** 10.3390/molecules23102631

**Published:** 2018-10-13

**Authors:** Zeyuan Wang, Jianfeng Cai, Qing Fu, Lingping Cheng, Lehao Wu, Weiyue Zhang, Yan Zhang, Yu Jin, Chunzhi Zhang

**Affiliations:** 1 School of Biological Engineering, Dalian Polytechnic University, Dalian 116034, China; wzydevil@126.com; 2 Engineering Research Center of Pharmaceutical Process Chemistry, Ministry of Education, School of Pharmacy, East China University of Science and Technology, Shanghai 200237, China; 921377549@163.com (J.C.); fuqing@ecust.edu.cn (Q.F.); clp0708@163.com (L.C.); 3 School of Pharmacy, Shanghai Jiao Tong University, Shanghai 200240, China; wulehaogo@sjtu.edu.cn (L.W.); zhangweiyue@sjtu.edu.cn (W.Z.)

**Keywords:** *Anemarrhena asphodeloides*, purification, anti-inflammation, N9

## Abstract

Fifteen unreported compounds in *Anemarrhena asphodeloides*, iriflophene (**3**), hostaplantagineoside C (**7**), tuberoside G (**8**), spicatoside B (**9**), platycodin D (**14**), platycoside A (**15**), platycodin D2 (**16**), polygalacin D2 (**17**), platycodin D3 (**18**), isovitexin (**20**), vitexin (**21**), 3,4-dihydroxyallylbenzene-3-*O*-α-l-rhamnopyranosyl(1→6)-β-d-glucopyranoside (**22**), iryptophan (**24**), adenosine (**25**), α-d-Glucose monoallyl ether (**26**), together with eleven known compounds (**1**, **2**, **4**–**6**, **10**–**13**, **19** and **23**), were isolated from the rhizomes of *Anemarrhena asphodeloides*. The chemical structures of these compounds were characterized using HRMS and NMR. The anti-inflammatory activities of the compounds were evaluated by investigating their ability to inhibit LPS-induced NO production in N9 microglial cells. Timosaponin BIII (TBIII) and trans-hinokiresinol (*t*-HL) exhibited significant inhibitory effects on the NO production in a dose-dependent manner with IC_50_ values of 11.91 and 39.08 μM, respectively. Immunoblotting demonstrated that TBIII and *t*-HL suppressed NO production by inhibiting the expressions of iNOS in LPS-stimulated N9 microglial cells. Further results revealed that pretreatment of N9 microglial cells with TBIII and t-HL attenuated the LPS-induced expression tumor necrosis factor (TNF)-α and interleukin-6 (IL-6) at mRNAs and protein levels. Moreover, the activation of nuclear factor-κB (NF-κB) and phosphatidylinositol 3-kinase (PI3K)/Akt signaling pathways were inhibited by TBIII and *t*-HL, respectively. Our findings indicate that the therapeutic implication of TBIII and *t*-HL for neurogenerative disease associated with neuroinflammation.

## 1. Introduction

Microglia, the specialized macrophages, are the major cellular source and regulate cerebral inflammatory reactions in the central nervous system (CNS) [1]. They can be over-stimulated during neuropathological conditions and neurodegenerative disorders, including multiple sclerosis [2], Parkinson’s disease [3] and Alzheimer’s disease [4]. Although these diseases are complicated and difficult to cure, neuroinflammation plays an important role, and lines of evidence showed that blocking neuroinflammation could either delay the onset or alleviate the symptoms of these diseases [5,6]. Upon abnormal stimulation, microglia are activated with the release of pro-inflammatory mediators which further promote neuroinflammatory injury. The inductions of pro-inflammatory mediators including tumor necrosis factor (TNF)-α, interleukin-6 and nitric oxide (NO), are indicators of microglial activation. Therefore, the inhibition of NO production or the pro-inflammatory cytokines is considered a strategy for the treatment and prevention of inflammatory conditions and related diseases [7]. Phosphatidylinositol 3-kinase (PI3K)/Akt, and nuclear factor-κB (NF-κB) are the upstream signaling pathways to regulate the expressions of inflammatory mediators in microglia [8,9]. Thus, to counter the regulatory mechanisms is essential for avoiding the escalation of the inflammatory process in CNS. Collectively, this information provides the possibility to identify agents that target over-stimulated microglial cells and to determine their anti-inflammatory mechanism.

Chemical components derived from natural products have attracted considerable attention in the field of disease and health, and diverse compounds from natural products have been reported to be effective in controlling microglial activation [10,11,12]. *Anemarrhena asphodeloides* (*A. asphodeloides*) belonging to traditional Chinese medicines, has been commonly used for therapeutic purposes for thousands of years. Previous reports on the phytochemical studies on this plant revealed various types of secondary metabolites, such as saponins, flavonoids, phenylpropanoids and alkaloids [13]. Saponins are the main ingredients of *A. asphodeloides* and are responsible for its biological activities [14,15]. Saponins in *A. asphodeloides* could significantly inhibit the inflammatory mediators production in Aβ_25–35_ stimulated macrophages [16]. Timosaponin BII, a main active saponin isolated from *A. asphodeloides* has been reported to ameliorate scopolamine-induced cognition deficits by attenuating acetylcholinesterase activity and brain oxidative damage in mice [17]. Therefore, saponins in *A. asphodeloides* might be the source for searching novel neuroactive compounds. In the present study, we aim to purify the saponins-enrich part of *A. asphodeloides* extract and evaluate the anti-neuroinflammatory activities of the isolated compounds. The underlying mechanisms of the active components will be further investigated.

## 2. Results and Discussion

### 2.1. Purification and Identification of Chemical Constituents

**Figure 1:**
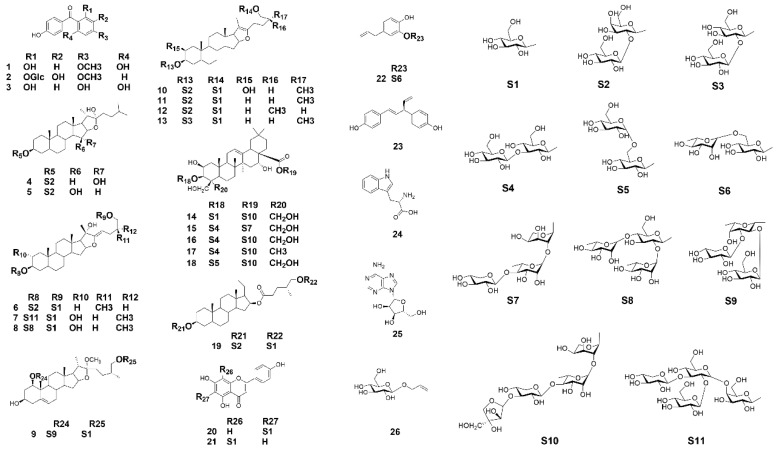
Structures of the isolated compounds from *A. asphodeloides*.

A systematic phytochemical investigation of *A. asphodeloides* was carried out using multiple chromatographic methods. The saponins-rich part of *A. asphodeloides* was obtained by a novel PGRP stationary phase. The subsequent off-line 2D-HILIC/RPLC system with high orthogonality gave rise to the isolation of twenty-six compounds including fifteen undescribed compounds (**3**, **7**–**9**, **14**–**18**, **20**–**22** and **24**–**26**) in *A. asphodeloides*. The flowchart of preparation and isolation of saponins-rich part was shown in Figure S1. All compounds were characterized by the determination of purity, HRMS and NMR data. By comparison of their spectroscopic data with those reported in the literature, the chemical structures of the isolated compounds were established as 2,6,4′-trihydroxy-4-methoxybenzophenone (**1**), Zimoside A (**2**), Iriflophene (**3**), Anemarrhenasaponin II (**4**), Anemarrhenasaponin I (**5**), Anemarnoside B (**6**), Hostaplantagineoside C (**7**), Tuberoside G (**8**), Spicatoside B (**9**), Timosaponin D (**10**), Timosaponin BIII (**11**), Macrostemonoside F (**12**), Timosaponin C (**13**), Platycodin D (**14**), Platycoside A (**15**), Platycodin D2 (**16**), Polygalacin D2 (**17**), Platycodin D3 (**18**), Anemarnoside A (**19**), Isovitexin (**20**), Vitexin (**21**), 3,4-dihydroxyallylbenzene-3-O-α-l-rhamnopyranosyl(1→6)-β-d-glucopyranoside (**22**), *trans*-Hinokiresinol (**23**), Tryptophan (**24**), Adenosine (**25**) and α-d-Glucose monoallyl ether (**26**) [18,19,20,21,22,23]. Structures of these isolated compounds were shown in Figure 1 and detailed structure characterizations were described in Supporting Information.

### 2.2. Cytotoxic Effects of the Purified Constituents against N9 Microglial Cells

To evaluate the cytotoxicities of the twenty-six purified compounds against N9 microglial cells, MTT assay was performed. As shown in Table 1, most of the compounds are non-cytotoxic, but three compounds (**4**, **14**, **18**) dramatically reduced cell viability to 60% or lower at 25 and 50 μM. According to the previous report, platycodin D (**14**) and platycodin D3 (**18**) showed significant cytotoxicity towards various kinds of tumor cells [24,25,26,27]. Very limited information regarding anemarrhenasaponin II (**4**) was available from publications. The findings of relatively high cytotoxicity of anemarrhenasaponin II (**4**) against N9 microglial cells are the first to be discovered and reported in this study.

Interesting observation is that compound **4** and **5** are optical isomers, while compound **4** but not compound **5** exhibited potent cytotoxicity.

### 2.3. Inhibitory Effects of the Purified Constituents on Lps-Stimulated NO Production in N9 Microglial Cells

Microglial cells can be activated by lipopolysaccharide (LPS) to produce NO and a variety of inflammatory cytokines including TNF-α and IL-6, which contribute to the pathogenesis of neural damage in many CNS disorders [28]. Therefore, LPS-stimulated N9 microglial cells provide us with an excellent model for investigating neuroinflammatory response and subsequently for screening anti-inflammatory drugs and their underlying mechanisms.

**Figure 2:**
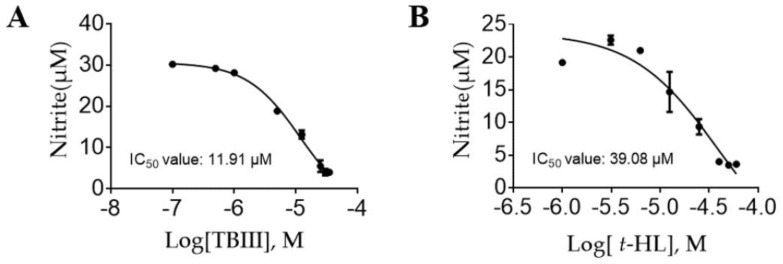
Inhibitory effects of TBIII (**A**) and *t*-HL (**B**) in LPS-stimulated N9 microglial cells. Cells were pretreated with different concentrations of TBIII and *t*-HL 1 h prior to the addition of LPS (100 ng/mL) and were further incubated for 24 h. NO production was determined by using the Griess reagent. IC_50_ values and curve fitting were determined using Graphpad Prism (GraphPad, Avenida, CA, USA). Data represent mean ± S.E.M.

In this study, we used LPS to activate mouse N9 microglia and investigated the inhibitory effects of the isolated compounds on NO production. Parthenolide (PAR) was used as a positive control in the assay. As shown in Table 1, among the twenty-six isolated compounds, four compounds (**4**, **11**, **18**, **23**) significantly inhibited LPS-induced NO production by more than 50% at 25 μM, and even higher at 50 μM. Considering the results of cytotoxicity test, the significant NO reduction caused by compound **4** and **18** may be at least partly contributed by cell death due to their high cytotoxicities. Notably, the non-cytotoxic compound **11** (*Timosaponin BIII*, TBIII) and compound **23** (*trans*-Hinokiresinol, *t*-HL) at 50 μM showed significant NO inhibition of 86% and 83%, respectively. Interestingly, compounds **11** and **13** are enantiomers with similar structures, but compound **13** exhibited much weaker inhibitory activity. Dose-effect curves of TBIII and *t*-HL were further determined and IC_50_ values were obtained as 11.91 and 39.08 μM, respectively (Figure 2A,B). According to previous reports, TBIII exhibited antidepressive activity in a mouse model of postpartum depression [29] and *t*-HL exerted protective effect on neuronal injury [19], which were in line with our results. Our findings implied that these two compounds could be effective anti-neuroinflammatory agents. Therefore, TBIII and *t*-HL were chosen for subsequent detailed study.

### 2.4. Effects of TBIII and t-HL on iNOS Expression in LPS-Stimulated N9 Microglial Cells

**Figure 3:**
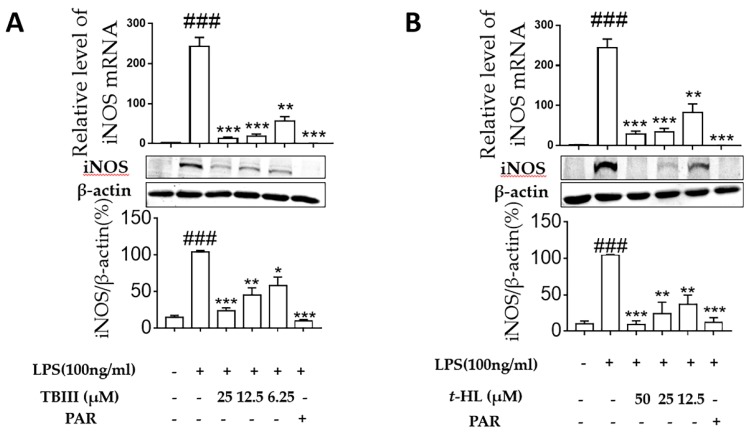
Effects of TBIII (**A**) and *t*-HL (**B**) on the expression of iNOS induced by LPS in N9 microglial cells. The iNOS protein levels were analyzed by Western blot. Cells were treated with different concentrations of TBIII and *t*-HL for 1 h and then stimulated with LPS (100 ng/mL) for 24 h. The iNOS signal was normalized to the β-actin signal. Parthenolide (PAR, 10 μM) was used as a positive control. The data were obtained from three independent experiments and expressed as the means ± S.E.M. * *p* < 0.05, ** *p* < 0.01, *** *p* < 0.001 versus the LPS-treated group; ^###^ *p* < 0.001 versus the control group.

To investigate whether the inhibitory effects of TBIII and *t*-HL on NO production were mediated through inhibition of corresponding inducible nitric oxide synthase (iNOS) modulation, western blotting was performed to determine iNOS expression levels in N9 microglial cells. As shown in Figure 3, the protein expression of iNOS were markedly up-regulated in response to LPS, and were significantly attenuated by TBIII and *t*-HL in dose-dependent manners (Figure 3A,B), respectively. These findings supported the observation that TBIII and *t*-HL inhibited NO production in LPS-stimulated N9 microglia and indicated that these two compounds reduced NO production by suppressing iNOS expression.

### 2.5. Effects of TBIII and t-HL on Inflammatory Cytokines in LPS-Stimulated N9 Cells

**Figure 4:**
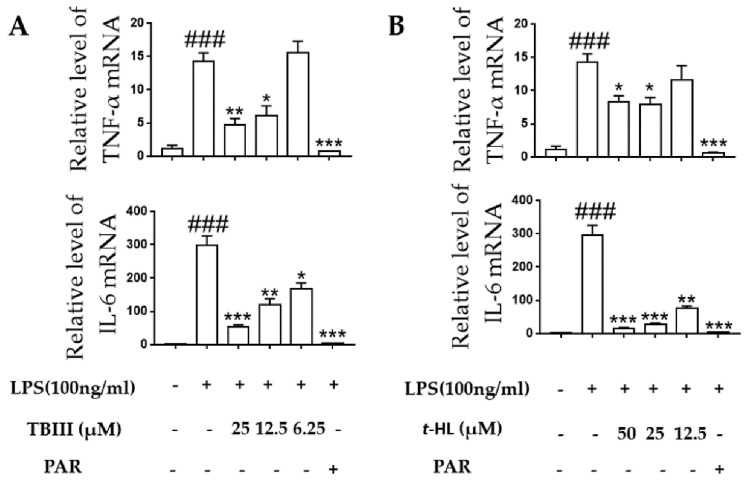
Effects of TBIII (**A**) and *t*-HL (**B**) on LPS-induced mRNA expression of pro-inflammatory cytokines in N9 microglial cells. Cells were treated with different concentrations of TBIII and *t*-HL for 1 h and then stimulated with LPS (100 ng/mL) for 4 h. TNF-α and IL-6 mRNA levels were determined by real-time PCR analysis. PAR (10 μM) was used as a positive control. The data were obtained from three independent experiments and expressed as the means ± S.E.M. * *p* < 0.05, ** *p* < 0.01, *** *p* < 0.001 versus the LPS-treated group; ^###^ *p* < 0.001 versus the control group.

**Figure 5:**
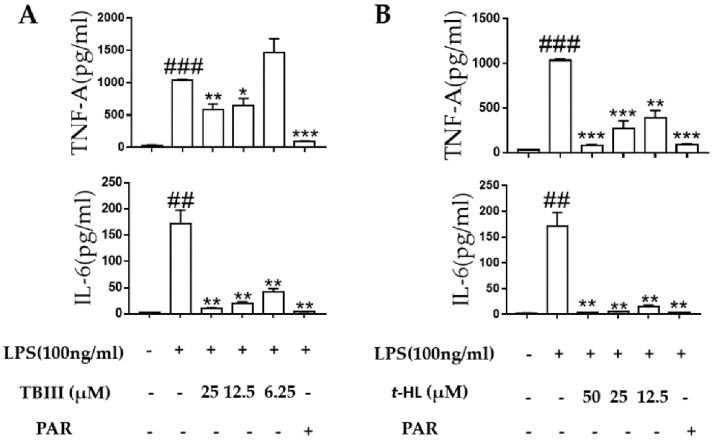
Effects of TBIII (**A**) and *t*-HL (**B**) on LPS-induced production of pro-inflammatory cytokines in N9 microglial cells. Cells were pre-treated with TBIII and *t*-HL for 1 h and then stimulated with LPS (100 ng/mL) for 24 h. The protein levels of TNF-α and IL-6 determined by ELISA according to manufacturer′s instructions. PAR (10 μM) was used as a positive control. The data were obtained from three independent experiments and expressed as the means ± S.E.M. * *p* < 0.05, ** *p* < 0.01, *** *p* < 0.001 versus the LPS-treated group; ^##^ *p* < 0.01, ^###^ *p* < 0.001 versus the control group.

The over-stimulated microglia produced pro-inflammatory cytokines including TNF-α and IL-6 during neuroinflammation which could then cause neuronal cell damage [30]. In the present study, the effects of TBIII and *t*-HL on LPS-induced production of these pro-inflammatory cytokines in N9 microglial cells were accessed by using RT-PCR and ELISA, respectively. As shown in Figure 4, exposure of N9 microglial cells to LPS for 4 h significantly up-regulated the mRNA expression of TNF-α and IL-6. Treatment of TBIII and *t*-HL significantly suppressed mRNA expression in a dose-dependent manner (Figure 4). Similar results were obtained at the protein level when N9 cells were treated with compounds before LPS stimulation (Figure 5). These data suggested that TBIII and *t*-HL alleviated LPS-stimulated dysregulation of inflammatory cytokines.

### 2.6. Effects of TBIII and t-HL on the Activation of Signaling Pathways in LPS-Stimulated N9 Microglial Cells

To investigate the anti-neuroinflammatory mechanisms of TBIII and *t*-HL, we identified two principle pathways NF-κB, and PI3K-Akt which are closely related to the inflammatory responses triggered by LPS in N9 microglial cells. NF-κB is a transcription factor that mediates immune and inflammatory responses. The activation of NF-κB involves the degradation of IKB-α, which binds to and inhibits NF-κB. The released NF-κB is then translocated into the nucleus to promote the expression of pro-inflammatory mediators [31]. Phosphatidylinositol 3-kinase (PI3K)/Akt signaling has been shown to be involved in the regulation of pro-inflammatory gene expression in microglia activated by LPS [32,33].

**Figure 6:**
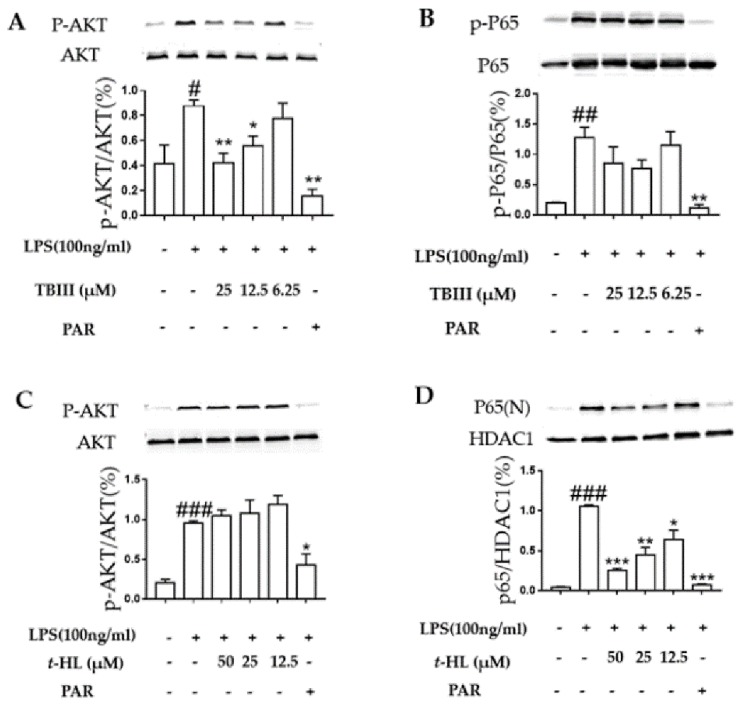
Effect of TBIII and *t*-HL on LPS-induced activation of Akt (**A,C**) and NF-κB (**B**,**D**) in N9 cells. Cells were treated with different concentrations of TBIII and *t*-HL for 1 h prior to stimulation with LPS (100 ng/mL). Nuclear and cytoplasmic proteins were obtained and analyzed by western blot. PAR (10 μM) was used as a positive control. The data shown represent the mean ± S.E.M of three independent experiments. * *p* < 0.05, ** *p* < 0.01, *** *p* < 0.001 versus the LPS-treated group; ^#^ *p* < 0.05, ^##^ *p* < 0.01, ^###^ *p* < 0.001 versus the control group.

Figure 6A illustrated that phosphorylation of Akt signaling protein was significantly increased with LPS treatment, and it was attenuated by TBIII without changing total Akt protein level. In contrast, TBIII exerted no effect on NF-κB signaling (Figure 6B). Our data suggest that one mechanism of the anti-inflammatory action of TBIII in microglia is the inhibition of the Akt signaling pathway.

As shown in Figure 6C, the activation of PI3/Akt signaling pathways were not affected by *t*-HL. However, *t*-HL significantly prevented LPS-induced NF-κB p65 subunit into nuclei dose-dependently (Figure 6D). Therefore, it suggested that *t*-HL-mediated inhibition of LPS-induced NF-κB activation might be one of the possible mechanisms underlying its inhibitory actions on iNOS and pro-inflammatory cytokines production by microglial cells.

Altogether, TBIII and *t*-HL exerted anti-neuroinflammatory activities through different mechanisms by inhibiting different signaling pathways.

## 3. Materials and Methods

### 3.1. Chemicals and Reagents

Activated carbon was purchased from Shanghai Activated Carbon Co., Ltd. (Shanghai, China). A reversed-phase (RP) stationary phase named CP and a positively charged reversed-phase (PGRP) stationary phase named RA3 (60 μm, 100 Å) were home-made based on previous reports [34,35]. C18 stationary phase (60 μm, 100 Å) was purchased from Acchrom Technologies Co., Ltd. (Beijing, China).

Preparative grade methanol and acetonitrile were purchased from Fulltime special solvent Co., Ltd. (Anhui, China). The water used in this study was purified with a Milli-Q water purification system (Millipore, Bedford, MA, USA).

The NO detection kit was obtained from Beyotime Institute of Biotechnology (Jiangsu, China). Iscove’s Modified Dulbecco’s Medium (IMDM) was purchased from HyClone (Logan, UT, USA). SDS-PAGE Sample Loading Buffer was purchased from Yeasen (Shanghai, China). TNF-α ELISA kit was obtained from eBioscience (San Diego, CA, USA). IL-6 ELISA kit was obtained from MultiSciences Biotechnology Corporate Limited (Lianke, Hangzhou, China). Antibodies were obtained as following: Anti-iNOS, P65, Akt, HDAC1, and phosphor-P65, Akt were purchased from Cell Signaling Technology (Danvers, MA, USA); anti-β-actin was purchased from Sigma Chemical Company (St. Louis, MO, USA). IRDye^®^800CW secondary antibodies were purchased from LICOR (Lincoln, NE, USA). Other chemicals were obtained from Sigma Chemical Company (St. Louis, MO, USA).

### 3.2. Apparatus

The preparative high-performance liquid chromatography (HPLC) was performed on a Waters Auto-Purification system, which consisted of a Waters 2545 binary gradient module, a Waters 2767 sample manager and a Waters 2489 UV/visible detector. Data were collected using a Masslynx 4.1 workstation (Waters, Milford, MA, USA)

The isolated compounds were identified by HRMS (Agilent, Palo Alto, CA, USA) and NMR (Bruker, Karlsruhe, Germany). The HRMS analysis was performed on Agilent 1290 Infinity LC/6530 Q-TOF MS (Agilent, Palo Alto, CA, USA). ^1^H-NMR spectra and ^13^ C-NMR spectra were measured on a BRUKER AVANCE III-500 spectrometer (^1^H-NMR at 500 MHz; ^13^ C-NMR at 125 MHz) with DMSO-d6 as the solvent.

### 3.3. Plant Materials

The rhizomes of *A. asphodeloides* were purchased from Lei Yun Shang Pharmaceutical store (Shanghai, China).

### 3.4. Preparation of Saponins-Rich part

The *A. asphodeloides* powder (17 kg) was extracted twice with ethanol for 1 h. Filtrate of twice times was gathered and 0.4% activated carbon (g/mL) was added for decolorization. The discolored filtrate was concentrated by rotary evaporation (Chengxian, Shanghai, China) at 55 °C under reduced pressure. The residue was dissolved in 5 L of water for solid phase extraction (SPE) with the cartridge packing CP, successively eluted using water and ethanol with three column volumes each time. The ethanol eluate was concentrated by vacuum distillation (Chengxian, Shanghai, China) to yield 420 g of residue (called crude extract below). 125 g of crude extract were dissolved in 2.5 L of methanol for further isolation using a preparative dynamic axial compression (DAC) column packed with 300 g C18 stationary phase (50 × 250 mm, i.d., 60 μm) with a gradient elution, which yielded a polar part of 12.8 g, a medium polar part of 92.1 g and a weak polar part of 7.9 g. The medium polar part was selectively separated into a xanthones-rich part (62 g) and a saponins-rich part (25 g) by using a preparative RA3 column (20 × 250 mm, i.d., 60 μm) with simple pH adjusting.

### 3.5. Purification of Compounds from the Saponins-Rich Part

As shown in Figure S1, the saponins-rich part (12 g) was first fractionated by a preparative XAmide column (20 × 250 mm, i.d., 10 μm, Acchrom, Beijing, China) using a gradient of acetonitrile: water (95:5 to 60:40, *v*/*v*), to afford seven fractions (Fr.1 to Fr.7) according to a manual method. Fr.1 (0.9 g) was subsequently chromatographed on a semi-preparative C18ME column (10 × 250 mm, i.d., 10 μm, Acchrom, Beijing, China) using a gradient solvent system of acetonitrile: water (10:90 to 90:10, *v*/*v*) to give compounds 1 (154.7 mg, 99.9%), 3 (6.4 mg, 96.9%), 4 (27.8 mg, 96.9%) and 23 (6.1 mg, 95.4%). Fr.6 (1.2 g) was repeated on a C18ME column (10 × 250 mm, i.d., 10 μm) with a stepwise gradient of acetonitrile: water (5:95 to 55:45, *v*/*v*) resulted in the isolation of compounds 2 (6.0 mg, 99.9%), 20 (6.6 mg, 99.9%), 21 (7.6 mg, 99.9%), 22 (10 mg, 95.9%), 25 (7.5 mg, 99.5%), 26 (2.0 mg, 99.5%). Fr.7 (6.4 g) was subjected to C18ME RPLC (20 × 250 mm, i.d., 10 μm) using a gradient program of acetonitrile: water (15:85 to 58:42, *v*/*v*) to yield thirteen sub-fractions (Fr.7-1 to Fr.7-13). The sub-fractions were further purified over a semi-preparative C18ME column (10 × 250 mm, i.d., 10 μm) to obtain compounds **5** (22.1 mg, 95.2%), **6** (89.2 mg, 96.9%), **7** (49.6 mg, 96.9%), **8** (16.7 mg, 95.5%), **9** (98.1 mg, 97.8%), **10** (36.4 mg, 97.3%), **11** (58.4 mg, 98.0%), **12** (104.2 mg, 95.2%), **13** (136.0 mg, 96.4%), **14** (159.9 mg, 93.3%), **15** (59.6 mg, 95.8%), **16** (167.4 mg, 99.4%), **17** (45.6 mg, 97.0%), **18** (369.6 mg, 95.3%), **19** (40.8 mg, 96.8%), **24** (13.7 mg, 98.2%).

### 3.6. Cell Culture

The N9 microglial cell line was obtained from China Cell Line Bank. The cells were grown at 37 °C in IMDM supplemented with 10% heat-inactivated FBS under a humidified atmosphere of 5% CO_2_. In this experiment, cells were allowed to acclimate for 24 h before treatments.

### 3.7. MTT Assay for Cell Viability

Cell viability was evaluated by the MTT reduction assay [36]. Cells were seeded in 96-well plates at a density of 2 × 10^4^ cells/well and allowed to grow for 24 h before treatments. Thereafter, 100 μL medium containing various concentrations (0–50 μM) of compounds were added and incubated for another 24 h. MTT was dissolved in DD water (5 mg/mL) and 10 μL were added to each well. After 4 h, the medium was removed and 100 μL DMSO were filled in each well. The optical density was measured at 490 nm on a Flexstation 3 (Molecular Devices, Silicon Valley, CA, USA).

### 3.8. Bioassay for NO Production

Accumulation of nitrite, a metabolite of NO in the culture media, was measured by Griess assay as previously reported [37]. Briefly, cells were stimulated with 100 ng/mL of LPS for 24 h in the presence of the test compounds (0–50 μM). The cell culture supernatant (50 μL) was then reacted with isometric Griess reagent, and the release of NO was measured at 540 nm on a Flexstation 3 (Molecular Devices, Silicon Valley, CA, USA).

### 3.9. Total RNA Extraction and Real-Time PCR

Total RNA was extracted from cells using Trizol reagent (Invitrogen, Carlsbad, CA, USA). Reverse transcription of RNA was performed with the Reverse Transcription System A3500 kit (Promega, Madison, WI, USA) according to the manufacturer′s protocol. The complementary DNA (cDNA) was subsequently subjected to Real-Time PCR to quantify the transcripts of TNF-α and IL-6 using SYBR^®^ Green Real-time PCR Master Mix (TOYOBO, Osaka, Japan). The following primers were used: TNF-α (5′-TTC TCATTC CTG CTT GTG G-3′; 5′-ACT TGG TGG TTT GCT ACG-3′), IL-6(5′-GAG GAT ACC ACT CCC AAC AGA CC-3′; 5′-AAG TGC ATC ATCGTT GTT CAT ACA-3′). The primers for the mouse housekeeping gene glyceraldehyde-3-phosphate dehydrogenase (GAPDH) (5′-CCT TCC GTG TTC CTA CCC-3′; 5′-CAA CCT GGT CCTCAG TGT AG-3′). PCR was performed according to the following conditions: 94 °C for 3 min, 40 cycles of denaturation at 94 °C for 30 s, annealing at 56 °C for 45 s, extension at 72 °C for 30 s, followed by a final extension at 72 °C for 10 min. The quantified fold changes in messenger RNA (mRNA) in each sample were normalized to GAPDH expression and calculated using the 2exp (−ΔΔCt) method.

### 3.10. Measurements of TNF-α and IL-6 Production by ELISA

N9 cells were plated into 24-well plates (2 × 10^5^ cells/well). After cells became adherent, they were pretreated with different concentrations of TBIII and *t*-HL for 1 h before stimulation with 100 ng/mL of LPS. The supernatants of cultured medium were collected and the production of TNF-α and IL-6 was measured after incubation for 24 h using an ELISA kit according to manufacturer′s instructions.

### 3.11. Western Blot Analysis

N9 cells were plated into 12-well plates (4 × 10^5^ cells/well) and pretreated with different concentrations of TBIII and *t*-HL for 1 h, then stimulated by 100 ng/mL of LPS for 24 h. Nuclear and cytoplasmic proteins were obtained with nuclear and cytoplasmic protein extraction kit (Beyotime Biotechnology, Shanghai, China) according to the manufacturer′s protocol. Proteins were collected by SDS-PAGE Sample Loading Buffer (Yeasen, Shanghai, China) and heated at 99 °C for 10 min. Proteins in sample loading buffer were separated on 10% sodium dodecyl sulfate (SDS)-polyacrylamide gel electrophoresis (PAGE) and followed by transferring to nitrocellulose membranes (Whatman Protran, Dassel, Germany). The membranes were blocked with 5% non-fat milk for 1 h at room temperature, then incubated overnight at 4 °C with primary antibodies, followed by the respective IRDye^®^ 800CW secondary antibodies. The membranes were scanned using the 800 nm channel on an Odyssey^®^ CLX Infrared Imaging System. The immunoreactive bands were quantified using the NIH Image J software (NIH, Bethesda, MD, USA).

### 3.12. Statistical Analysis

The results are expressed as mean ± S.E.M. Data were analyzed by student′s test or one-way ANOVA as indicated *p* values < 0.05 were considered significant. All statistical analyses were performed with the GraphPad Software (GraphPad, Avenida, CA, USA).

## 4. Conclusions

In this study, we report here the results of our phytochemical and pharmacological investigation of the saponins-rich part from *A. asphodeloides*. This has results in the isolation and characterization of sixteen saponins and ten other compounds. Among them, compounds **3**, **7**–**9**, **14**–**18**, **20**–**22** and **24**–**26** were reported from *A. asphodeloides* for the first time. The anti-neuroinflammatory effects of the isolated compounds were evaluated. Two components (TBIII and *t*-HL) were significantly suppressed the production of NO and pro-inflammatory cytokines (TNF-α) and IL-6 by inhibiting the PI3K/Akt and NF-κB signaling pathways, respectively, in LPS-stimulated N9.

Our study indicates that TBIII and *t*-HL may contribute to the traditional therapeutic effects of *A. asphodeloides* in the traditional treatment of neurodegenerative disease and it is worth deeply exploring the druggability of these compounds.

## Figures and Tables

**Table 1:** Effects of isolated compounds on cell viability and NO production.

Compound	Name	% Cell Viability		Nitrite (μM)		% Inhibition of NO Production	
		50 μM	25 μM	50 μM	25 μM	50 μM	25 μM
1	*2,6,4′-Trihydroxy-4-methoxybenzo-phenone*	97.49 ± 1.85	98.73 ± 8.27	19.29 ± 0.10 *	26.78 ± 1.27 *	41.74 ± 3.90 *	15.35 ± 9.40
2	*Zimoside A*	94.62 ± 5.47	97.24 ± 3.27	21.41 ± 0.92 *	26.64 ± 1.13 *	32.77 ± 2.88 *	16.32 ± 3.55 *
3	*Iriflophene*	112.72 ± 5.32	107.03 ± 3.62	20.41 ± 0.38 *	26.06 ± 1.24 *	35.89 ± 1.20 *	18.14 ± 3.88 *
4	*Anemarrhena-saponin II*	8.57 ± 0.11 ^#^	63.92 ± 5.98 ^#^	3.80 ± 0.53 *	9.52 ± 0.84 *	87.98 ± 2.99 *	69.91 ± 4.86 *
5	*Anemarrhena-saponin I*	115.47 ± 6.83	110.42 ± 4.67	19.14 ± 0.59 *	29.82 ± 0.24 *	39.90 ± 1.85 *	6.36 ± 0.76 *
6	*Anemarnoside B*	104.33 ± 16.47	115.27 ± 5.52	26.38 ± 2.63	40.89 ± 2.04	17.23 ± 8.26	−28.28 ± 6.40
7	*Hostaplantagineo-side C*	79.71 ± 6.03	84.89 ± 1.82	23.52 ± 0.37 *	32.76 ± 0.85	25.75 ± 2.40 *	−3.57 ± 8.75
8	*Tuberoside G*	99.29 ± 2.46	112.82 ± 4.14	27.65 ± 0.74 *	35.05 ± 0.68 *	13.20 ± 2.32 *	−10.83 ± 2.14 *
9	*Spicatoside B*	94.29 ± 3.21	99.70 ± 3.13	24.20 ± 0.29 *	34.46 ± 0.41 *	24.01 ± 0.90 *	−8.22 ± 1.30 *
10	*Timosaponin D*	110.25 ± 2.57	111.11 ± 4.39	20.78 ± 2.00 *	29.22 ± 0.60	34.75 ± 6.27 *	8.57 ± 1.87
11	*Timosaponin BIII*	74.93 ± 3.15 ^#^	119.63 ± 11.75	4.24 ± 0.10 *	8.55 ± 0.25 *	86.67 ± 0.37 *	73.16 ± 0.80 *
12	*Macrostemono-side F*	105.77 ± 2.84	104.74 ± 3.16	11.82 ± 1.03 *	19.64 ± 1.21 *	62.89 ± 3.22 *	38.33 ± 3.80 *
13	*Timosaponin C*	97.19 ± 6.46	109.04 ± 4.85	13.36 ± 0.56 *	24.24 ± 1.32	58.10 ± 1.75 *	23.94 ± 4.13
14	*Platycodin D*	17.93 ± 1.99 ^#^	40.16 ± 9.18 ^#^	4.29 ± 0.59 *	28.94 ± 0.40 *	86.54 ± 2.71 *	8.65 ± 2.24
15	*Platycoside A*	108.08 ± 1.17	103.09 ± 3.81	22.55 ± 0.24 *	29.14 ± 1.22	29.19 ± 0.74 *	8.49 ± 3.84
16	*Platycodin D2*	101.05 ± 1.83	113.54 ± 4.62	24.94 ± 0.45 *	33.68 ± 1.44	21.67 ± 1.40 *	−5.78 ± 4.51
17	*Polygalacin D2*	88.00 ± 8.92	97.50 ± 1.83	26.21 ± 0.82 *	31.14 ± 0.78	17.74 ± 3.14 *	2.25 ± 2.46
18	*Platycodin D3*	9.07 ± 0.72 ^#^	9.23 ± 0.22 ^#^	3.50 ± 0.21 *	8.49 ± 0.36 *	89.01 ± 0.67 *	73.37 ± 1.14 *
19	*Anemarnside*	125.85 ± 3.00 ^#^	124.43 ± 3.13 ^#^	9.83 ± 0.30 *	23.63 ± 1.13 *	66.91 ± 2.82 *	25.23 ± 8.72 *
20	*Isovitexin*	95.68 ± 2.75	135.43 ± 11.78	22.82 ± 0.27 ^*^	28.39 ± 0.07 ^*^	29.77 ± 0.60 ^*^	10.21 ± 4.73
21	*Vitexin*	81.75 ± 4.38 ^#^	90.52 ± 2.51	17.81 ± 1.13 *	28.88 ± 1.38	44.11 ± 3.56 *	9.39 ± 4.33
22	*3,4-dihydroxyallyl-benzene-3-O-α-L-rhamnopyranosyl (1→6)-β-d-glucopy-ranoside*	95.17 ± 4.91	99.63 ± 5.82	19.43 ± 1.79 *	27.23 ± 0.21 *	38.97 ± 5.62 *	14.48 ± 0.65 *
23	*trans-Hinokiresinol*	98.29 ± 11.55	108.44 ± 4.39	5.18 ± 0.06 *	14.93 ± 0.41 *	83.73 ± 0.22 *	53.12 ± 1.30 *
24	*Tryptophan*	92.44 ± 8.71	104.52 ± 6.13	24.19 ± 0.72 *	30.49 ± 0.93	24.03 ± 2.27 *	4.24 ± 2.92
25	*Adenosine*	80.54 ± 6.19	81.56 ± 1.26	20.69 ± 1.74 *	27.43 ± 1.35	35.09 ± 5.47 *	13.93 ± 4.23
26	*α-d-Glucose-monoallyl ether*	105.47 ± 16.13	107.17 ± 0.63	22.60 ± 2.19 *	29.57 ± 0.71	29.03 ± 8.43 *	7.13 ± 2.23

The nitrite levels of control and LPS (100 ng/mL)-treated group were 4.60 ± 0.67 and 31.81 ± 0.41 μM, respectively. The data were obtained from three independent experiments and expressed as the means ± S.E.M. Data were analyzed by one-way ANOVA. * *p* < 0.05 vs. the LPS-treated group; # *p* < 0.05 vs. the control group.

## Supplementary Materials

The following are available online, Figure S1: Flowchart of the purification of saponin-rich part, Table S1: ^13^C NMR data of compounds 14–18.
