# Supplementary material for: Anti-Inflammatory Activities of Compounds Isolated from the Rhizome of Anemarrhena asphodeloides
Source: Molecules. 2018 Oct 13;23(10):2631. doi: 10.3390/molecules23102631 (PMC6222787; doi:10.3390/molecules23102631)
Supplement: Supplementary file 1 [file molecules-23-02631-s001.pdf]

Supplementary Materials

1. Flow Chart of Preparation and Isolation of Saponins-rich Part

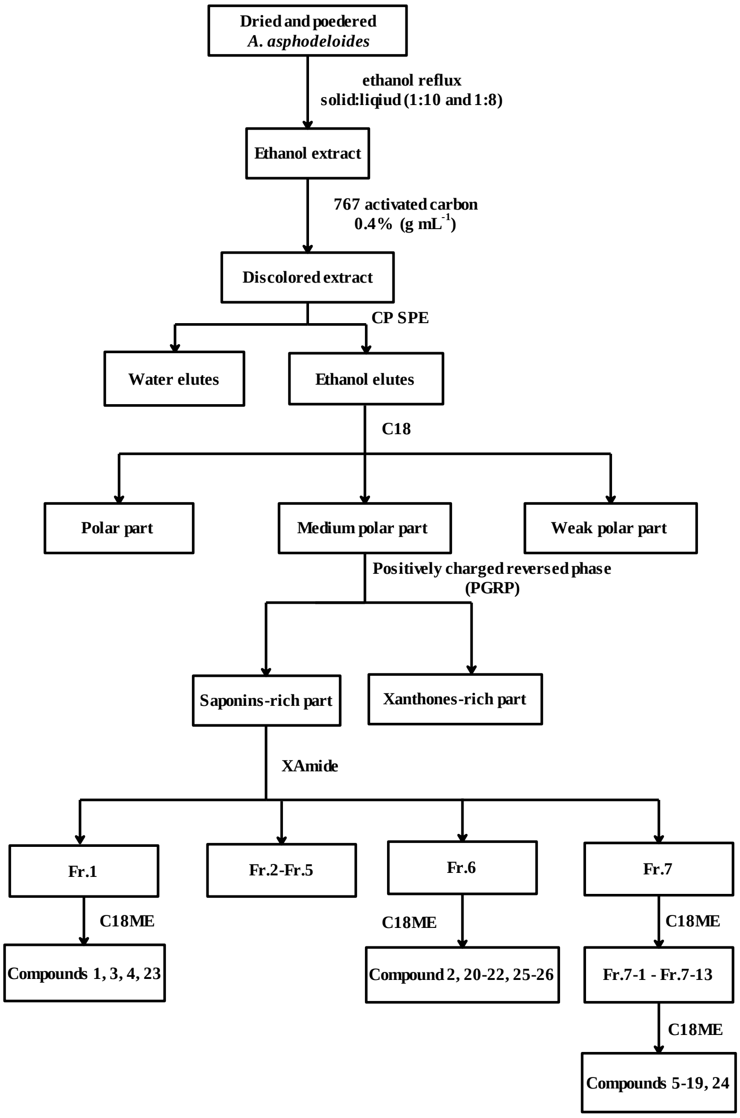

Figure S1. Flowchart of the purification of saponins-rich part.

2. Identification of compounds

2.1. Identification of known compounds

Compound 1

Name: 2, 6, 4'-trihydroxy-4-methoxybenzophenone

Molecular Formula： $C_{14}H_{12}O_5$ [M + H] +: 261.0759

^{1}H-NMR and ^{13}C-NMR spectroscopic data

^{1}H-NMR (500 MHz, DMSO-d_6)

7.57 (2H, *d*, *J* = 7.5 Hz, H-2′, H-6′), 6.80 (2H, *d*, *J* = 7.5 Hz, H-3′, H-5′), 5.94 (2H, *s*, H-5).

^{13}C-NMR (125 MHz, DMSO-d_6)

108.9 (C-1), 162.2 (C-2), 93.1 (C-3), 165.8 (C-4), 93.1 (C-5), 162.2 (C-6), 194.1 (C-7), 130.6 (C-1′), 132.0 (C-2′), 115.4 (C-3′), 157.6 (C-4′), 115.4 (C-5′), 132.0 (C-6′), 55.4 (-OCH_3).

Compound 2

Name: *Zimoside A*

Molecular Formula：$C_{20}H_{22}O_{10}$ [M + Na] $^+$: 445.1107

^{1}H-NMR and ^{13}C-NMR spectroscopic data

^{1}H-NMR (500 MHz, DMSO-d_6)

6.13 (1H, *d*, *J* = 1.5 Hz, H-5), 6.29 (1H, *d*, *J* = 1.5 Hz, H-5), 6.77 (2H, *d*, *J* = 7.0 Hz, H-3′, H-5′), 7.57 (2H, *d*, *J* = 7.0 Hz, H-2′, H-6′).

^{13}C-NMR (125 MHz, DMSO-d_6)

110.6 (C-1), 156.4 (C-2), 161.0 (C-3), 156.2 (C-4), 95.1 (C-5), 92.9 (C-6), 192.5 (C-7), 129.0 (C-1′), 131.8 (C-2′), 115.1 (C-3′), 161.0 (C-4′), 115.1 (C-5′), 131.8 (C-6′), 100.6 (C-1′′), 73.2 (C-2′′), 76.7 (C-3′′), 69.8 (C-4′′), 77.3 (C-5′′), 60.8 (C-6′′), 55.1 (-OCH_3).

Compound 4

Name: *Anemarrhenasaponin II*

Molecular Formula：$C_{39}H_{66}O_{14}$ [M + H] $^+$: 759.4541

^{1}H-NMR and ^{13}C-NMR spectroscopic data

^{1}H-NMR (500 MHz, DMSO-d_6)

4.90 (1H, *d*, *J* = 4.5 Hz, Gal-1′), 4.96 (1H, *d*, *J* = 3.5 Hz, H-15), 5.25 (1H, *d*, *J* = 3.0 Hz, H-16), 5.26 (1H, *d*, *J* = 3.5 Hz, Glc-1′′).

^{13}C-NMR (125 MHz, DMSO-d_6)

29.9 (C-1), 26.5 (C-2), 73.2 (C-3), 30.1 (C-4), 34.6 (C-5), 26.3 (C-6), 26.2 (C-7), 34.9 (C-8), 40.1 (C-9), 31.5 (C-10), 20.5 (C-11), 36.0 (C-12), 41.6 (C-13), 55.7 (C-14), 76.2 (C-15), 80.5 (C-16), 60.3 (C-17), 16.3 (C-18), 26.0 (C-19), 40.2 (C-20), 16.0 (C-21), 108.9 (C-22), 34.2 (C-23), 25.7 (C-24), 28.7 (C-25), 25.6 (C-26), 25.4 (C-27), 100.8 (Gal C-1′), 79.1 (C-2′), 70.0 (C-3′), 64.3 (C-4′), 73.9 (C-5′), 61.9 (C-6′), 103.9 (Glc C-1′′), 74.7 (C-2′′), 75.2 (C-3′′), 67.8 (C-4′′), 77.0 (C-5′′), 61.0 (C-6′′).

Compound 5

Name: *Anemarrhenasaponin I*

Molecular Formula：$C_{39}H_{66}O_{14}$ [M-H] $^-$: 757.4370

^{1}H-NMR and ^{13}C-NMR spectroscopic data

^{1}H-NMR (500 MHz, DMSO-d_6)

4.96 (1H, *br s*, Gal-1′), 5.09 (1H, *s*, H-15), 5.24 (1H, *br s*, H-16), 5.26 (1H, *br s*, Glc-1′′).

^{13}C-NMR (125 MHz, DMSO-d_6)

29.9 (C-1), 26.3 (C-2), 73.9 (C-3), 30.3 (C-4), 35.4 (C-5), 26.0 (C-6), 25.9 (C-7), 35.9 (C-8), 40.1 (C-9), 32.7 (C-10), 20.4 (C-11), 36.3 (C-12), 40.4 (C-13), 59.9 (C-14), 77.7 (C-15), 89.4 (C-16), 59.9 (C-17), 17.4 (C-18), 23.8 (C-19), 40.4 (C-20), 15.9 (C-21), 109.1 (C-22), 34.6 (C-23), 23.8 (C-24), 27.9 (C-25), 22.7 (C-26), 22.6 (C-27), 100.8 (Gal C-1′), 79.1 (C-2′), 73.2 (C-3′), 67.8 (C-4′), 74.7 (C-5′), 60.3 (C-6′), 103.9 (Glc C-1′′), 75.2 (C-2′′), 76.2 (C-3′′), 70.0 (C-4′′), 79.1 (C-5′′), 61.1 (C-6′′).

Compound 6

Name: *Anemarnoside B*

Molecular Formula：$C_{45}H_{74}O_{19}$ [M-H] $^-$: 917.4736

^{1}H-NMR and ^{13}C-NMR spectroscopic data

^{1}H-NMR (500 MHz, DMSO-d_6)

4.66 (1H, *m*, Glu-1′′′), 4.92 (1H, *br s*, Gal-1′), 4.97 (1H, *br s*, Glc-1′′), 5.26 (1H, *br s*, H-16).

^{13}C-NMR (125 MHz, DMSO-d_6)

29.9 (C-1), 26.4 (C-2), 73.6 (C-3), 30.2 (C-4), 36.0 (C-5), 26.4 (C-6), 26.4 (C-7), 34.0 (C-8), 40.1 (C-9), 34.6 (C-10), 17.3 (C-11), 38.2 (C-12), 43.3 (C-13), 54.2 (C-14), 34.2 (C-15), 83.5 (C-16), 63.9 (C-17), 11.5 (C-18), 26.0 (C-19), 74.8 (C-20), 20.7 (C-21), 152.8 (C-22), 100.7 (C-23), 29.8 (C-24), 34.6 (C-25), 73.8 (C-26), 14.2 (C-27), 103.3 (Gla C-1′), 79.1 (C-2′), 73.3 (C-3′), 67.9 (C-4′), 74.3 (C-5′), 60.3 (C-6′), 104.7 (Glc C-1′′), 75.2 (C-2′′), 76.2 (C-3′′), 70.0 (C-4′′), 76.9 (C-5′′), 61.1 (C-6′′), 103.8 (Glc C-1′′′), 73.6 (C-2′′′), 77.1 (C-3′′′), 70.1 (C-4′′′), 76.9 (C-5′′′), 62.2 (C-6′′′).

Compound 10

Name: *Timosaponin D*

Molecular Formula：$C_{45}H_{74}O_{19}$ [M-H]$^-$: 917.4739

^{1}H-NMR and ^{13}C-NMR spectroscopic data

^{1}H-NMR (500 MHz, DMSO-d_6)

4.67 (1H, *m*, Glc-1′′′), 4.91 (1H, *br s*, Glc-1′), 4.91 (1H, *br s*, Glc-1′′), 5.26 (1H, *br s*, H-16).

^{13}C-NMR (125 MHz, DMSO-d_6)

40.1 (C-1), 63.9 (C-2), 79.1 (C-3), 29.9 (C-4), 36.0 (C-5), 26.0 (C-6), 26.4 (C-7), 34.6 (C-8), 41.1 (C-9), 38.2 (C-10), 20.7 (C-11), 40.1 (C-12), 43.3 (C-13), 54.2 (C-14), 29.8 (C-15), 83.5 (C-16), 62.2 (C-17), 14.2 (C-18), 23.6 (C-19), 103.3 (C-20), 11.4 (C-21), 152.8 (C-22), 34.0 (C-23), 30.2 (C-24), 73.8 (C-26), 17.3 (C-27), 100.7 (Glc C-1′), 77.0 (C-2′), 73.6 (C-3′), 67.8 (C-4′), 74.8 (C-5′), 60.3 (C-6′), 104.6 (Glc C-1′′), 74.3 (C-2′′), 75.2 (C-3′′), 70.0 (C-4′′), 76.8 (C-5′′), 61.1 (C-6′′), 103.8 (Glc C-1′′′), 73.3 (C-2′′′), 76.9 (C-3′′′), 70.1 (C-4′′′), 76.2 (C-5′′′), 61.1 (C-6′′′).

Compound 11

Name: *Timosaponin BIII*

Molecular Formula：$C_{45}H_{74}O_{18}$ [M-H]$^{-}$: 901.4783

^{1}H-NMR and ^{13}C-NMR spectroscopic data

^{1}H-NMR (500 MHz, DMSO-d_6)

4.93 (1H, *m*, Gal-1′), 4.92 (1H, *m*, Glc-1′′), 5.26 (1H, *m*, Glc-1′′′).

^{13}C-NMR (125 MHz, DMSO-d_6)

28.6 (C-1), 26.0 (C-2), 73.2 (C-3), 28.8 (C-4), 36.0 (C-5), 26.3 (C-6), 25.6 (C-7), 34.5 (C-8), 40.1 (C-9), 34.7 (C-10), 21.9 (C-11), 42.5 (C-12), 43.3 (C-13), 54.2 (C-14), 29.9 (C-15), 83.6 (C-16), 64.6 (C-17), 16.8 (C-18), 24.5 (C-19), 102.9 (C-20), 11.5 (C-21), 151.5 (C-22), 33.9 (C-23), 23.6 (C-24), 32.6 (C-25), 73.5 (C-26), 14.1 (C-27), 100.7 (Gal C-1′), 79.1 (C-2′), 75.2 (C-3′), 67.8 (C-4′), 74.0 (C-5′), 60.3 (C-6′), 103.8 (Glc C-1′′), 74.8 (C-2′′), 77.0 (C-3′′), 70.0 (C-4′′), 76.9 (C-5′′), 61.1 (C-6′′), 103.3 (Glc C-1′′′), 73.8 (C-2′′′), 76.8 (C-3′′′), 70.1 (C-4′′′), 76.2 (C-5′′′), 61.1 (C-6′′′).

Compound 12

Name: *Macrostemonoside F*

Molecular Formula：$C_{45}H_{74}O_{18}$ [M + H] $^{+}$: 903.4946

^{1}H-NMR and ^{13}C-NMR spectroscopic data

^{1}H-NMR (500 MHz, DMSO-d_6)

0.63 (3H, *s*, H-18), 0.91 (3H, *s*, H-19), 0.92 (3H, *s*, H-27), 1.56 (3H, *s*, H-21), 4.94 (1H, *br s*, Glc-1′), 4.99 (1H, *br s*, Glc-1′′), 5.28 (1H, *br s*, Glc-1′′′).

^{13}C-NMR (125 MHz, DMSO-d_6)

30.2 (C-1), 26.4 (C-2), 73.3 (C-3), 30.6 (C-4), 36.0 (C-5), 30.0 (C-6), 26.0 (C-7), 34.7 (C-8), 39.5 (C-9), 34.6 (C-10), 20.7 (C-11), 40.1 (C-12), 43.3 (C-13), 54.3 (C-14), 30.7 (C-15), 83.6 (C-16), 63.7 (C-17), 16.8 (C-18), 23.7 (C-19), 102.9 (C-20), 11.6 (C-21), 151.6 (C-22), 33.9 (C-23), 22.8 (C-24), 32.6 (C-25), 73.5 (C-26), 14.1 (C-27), 100.7 (Glc C-1′), 79.1 (C-2′), 75.2 (C-3′), 67.8 (C-4′), 74.8 (C-5′), 60.3 (C-6′), 103.8 (Glc C-1′′), 74.0 (C-2′′), 77.0 (C-3′′), 70.0 (C-4′′), 76.9 (C-5′′), 61.1 (C-6′′), 103.3 (Glc C-1′′′), 73.8 (C-2′′′), 76.9 (C-3′′′), 70.1 (C-4′′′), 76.2 (C-5′′′), 61.2 (C-6′′′).

Compound 13

Name: *Timosaponin C*

Molecular Formula：$C_{45}H_{74}O_{18}$ [M + H] $^{+}$: 903.4968

^{1}H-NMR and ^{13}C-NMR spectroscopic data

^{1}H-NMR (500 MHz, DMSO-d_6)

0.60 (3H, *s*, H-18), 0.87 (3H, *d*, J = 6.6 Hz, H-19), 1.54 (3H, *s*, H-21), 4.91 (1H, *br s*, Glc-1′), 4.91 (1H, *br s*, Glc-1′′), 5.25 (1H, *br s*, Glc-1′′′).

^{13}C-NMR (125 MHz, DMSO-d_6)

29.9 (C-1), 26.4 (C-2), 73.3 (C-3), 30.2 (C-4), 36.0 (C-5), 26.0 (C-6), 26.3 (C-7), 34.7 (C-8), 39.4 (C-9), 34.6 (C-10), 20.7 (C-11), 40.1 (C-12), 43.3 (C-13), 54.3 (C-14), 30.6 (C-15), 83.6 (C-16), 63.7 (C-17), 14.1 (C-18), 23.7 (C-19), 102.9 (C-20), 11.6 (C-21), 151.6 (C-22), 33.9 (C-23), 22.8 (C-24), 32.6 (C-25), 73.5 (C-26), 16.8 (C-27), 100.7 (Glc C-1′), 79.1 (C-2′), 76.9 (C-3′), 67.8 (C-4′), 75.2 (C-5′), 61.1 (C-6′), 103.8 (Glc C-1′′), 74.8 (C-2′′), 73.8 (C-3′′), 70.0 (C-4′′), 77.0 (C-5′′), 60.3 (C-6′′), 103.3 (Glc C-1′′′), 74.0 (C-2′′′), 76.8 (C-3′′′), 70.1 (C-4′′′), 76.2 (C-5′′′), 61.1 (C-6′′′).

Compound 19

Name: *Anemarnoside A*

Molecular Formula：$C_{45}H_{76}O_{19}$ [M + Na] $^{+}$: 943.4873

^{1}H-NMR and ^{13}C-NMR spectroscopic data

^{1}H-NMR (500 MHz, DMSO-d_6)

4.90 (1H, *br s*, Glc-1′′′), 4.93 (1H, *br s*, Gal-1′), 4.96 (1H, *br s*, Glc-1′′), 5.25 (1H, *br s*, H-16).

^{13}C-NMR (125 MHz, DMSO-d_6)

30.1 (C-1), 26.4 (C-2), 73.9 (C-3), 30.0 (C-4), 36.0 (C-5), 26.0 (C-6), 26.0 (C-7), 34.8 (C-8), 40.1 (C-9), 34.6 (C-10), 20.2 (C-11), 37.8 (C-12), 41.9 (C-13), 53.4 (C-14), 34.8 (C-15), 73.3 (C-16), 56.4 (C-17), 56.4 (C-18), 13.3 (C-19), 23.7 (C-19), 16.7 (C-20), 12.8 (C-21), 172.7 (C-22), 31.6 (C-23), 28.4 (C-24), 32.6 (C-25), 73.5 (C-26), 16.5 (C-27), 100.8 (Gal C-1′), 79.1 (C-2′), 73.5 (C-3′), 67.8 (C-4′), 74.8 (C-5′), 60.3 (C-6′), 103.8 (Glc C-1′′), 75.2 (C-2′′), 76.2 (C-3′′), 70.0 (C-4′′), 76.9 (C-5′′), 61.1 (C-6′′), 103.2 (Glc C-1′′′), 73.8 (C-2′′′), 77.1 (C-3′′′), 70.2 (C-4′′′), 76.8 (C-5′′′), 61.2 (C-6′′′).

Compound 23

Name: *trans-Hinokiresinol*

Molecular Formula：$C_{17}H_{16}O_2$ [M-H] $^{-}$: 251.1077

^{1}H-NMR and ^{13}C-NMR spectroscopic data

^{1}H-NMR (500 MHz, DMSO-d_6)

7.11 (2H, *d*, *J* = 7.0 Hz, H-2′, H-6′), 7.01 (2H, *d*, *J* = 7.0 Hz, H-2′′, H-6′′), 6.74 (2H, *d*, *J* = 7.0 Hz, H-3′, H-5′), 6.71 (2H, *d*, *J* = 7.0 Hz, H-3′′, H-5′′), 6.43 (1H, *d*, *J* = 10.0 Hz, H-1).

^{13}C-NMR (125 MHz, DMSO-d_6)

127.5 (C-1), 130.7 (C-2), 46.6 (C-3), 141.3 (C-4), 114.3 (C-5), 128.2 (C-1′), 129.7 (C-2′), 115.2 (C-3′), 156.5 (C-4′), 115.2 (C-5′), 129.7 (C-6′), 133.3 (C-1′′), 128.4 (C-2′′), 115.4 (C-3′′), 155.4 (C-4′′), 115.4 (C-5′′), 128.4 (C-6′′).

1.2.2 Identification of unreported compounds

Compound 3: white powder, HRMS *m/z* 269.0429 [M + Na] $^{+}$ (calcd for $C_{13}H_{10}O_5$, 246.0528). ^{1}H-NMR (500 MHz, DMSO-d_6): δ 5.81 (2H, *s*, H-3, H-5), 6.78 (2H, *d*, *J* = 7.5 Hz, H-3′, H-5′), 7.54 (2H, *d*, *J* = 8.0 Hz, H-2′, H-6′). ^{13}C-NMR (125 MHz, DMSO-d_6): δ 106.6 (C-1), 160.4 (C-2), 94.3 (C-3), 161.6 (C-4), 94.3 (C-5), 160.4 (C-6), 194.3 (C-7), 131.5 (C-1′), 130.6 (C-2′), 114.7 (C-3′), 158.0 (C-4′), 114.7 (C-5′), 130.6 (C-6′).

Compound 7: white powder, HRMS *m/z* 1251.5603 [M + Na] $^+$ (calcd for $C_{56}H_{92}O_{29}$, 1228.5724). ^{1}H-NMR (500 MHz, DMSO-*d*$_6$): *δ* 4.93 (2H, *br s*, Gal-1′, Glc-1′′′′′), 5.07 (1H, *br s*, Glc-1′′), 5.26 (1H, *br s*, Xyl-1′′′′), 5.53 (1H, *br s*, Glc-1′′′). ^{13}C-NMR (125 MHz, DMSO-*d*$_6$): *δ* 45.0 (C-1), 69.5 (C-2), 83.1 (C-3), 33.7 (C-4), 43.9 (C-5), 28.6 (C-6), 32.9 (C-7), 34.3 (C-8), 53.7 (C-9), 36.0 (C-10), 21.1 (C-11), 38.9 (C-12), 40.1 (C-13), 56.1 (C-14), 33.7 (C-15), 83.1 (C-16), 67.8 (C-17), 13.3 (C-18), 13.2 (C-19). 75.7 (C-20), 23.6 (C-21), 162.4 (C-22), 90.1 (C-23), 30.1 (C-24), 34.5 (C-25), 73.5 (C-26), 17.2 (C-27), 100.7 (Gal C-1′), 73.3 (C-2′), 74.8 (C-3′), 79.1 (C-4′), 75.2 (C-5′), 60.3 (C-6′), 103.4 (Glc C-1′′), 79.4 (C-2′′), 85.1 (C-3′′), 69.9 (C-4′′), 76.2 (C-5′′), 61.1 (C-6′′), 103.4 (Glc C-1′′′), 73.5 (C-2′′′), 76.8 (C-3′′′), 70.0 (C-4′′′), 76.8 (C-5′′′), 61.0 (C-6′′′), 103.9 (Xyl C-1′′′′), 74.0 (C-2′′′′), 77.0 (C-3′′′′), 70.0 (C-4′′′′), 66.7 (C-5′′′′), 103.4 (Glc C-1′′′′′), 73.8 (C-2′′′′′), 76.8 (C-3′′′′′), 70.1 (C-4′′′′′), 76.9 (C-5′′′′′), 61.1 (C-6′′′′′).

Compound 8: white powder, HRMS *m/z* 1065.5471 [M + H] $^+$ (calcd for $C_{51}H_{84}O_{23}$, 1064.5403). ^{1}H-NMR (500 MHz, DMSO-*d*$_6$): *δ* 4.74 (1H, *m*, Glc-1′′), 4.93 (2H, *br s*, Glc-1′, Rha-1′′′′), 5.83 (1H, *br s*, Rha-1′′′). ^{13}C-NMR (125 MHz, DMSO-*d*$_6$): *δ* 44.9 (C-1), 70.0 (C-2), 83.5 (C-3), 34.0 (C-4), 43.3 (C-5), 28.6 (C-6), 30.4 (C-7), 34.6 (C-8), 54.0 (C-9), 36.3 (C-10), 21.1 (C-11), 38.2 (C-12), 40.5 (C-13), 55.8 (C-14), 33.7 (C-15), 83.1 (C-16), 67.8 (C-17), 13.3 (C-18), 11.5 (C-19), 76.8 (C-20), 23.5 (C-21), 162.4 (C-22), 90.2 (C-23), 29.8 (C-24), 35.5 (C-25), 75.7 (C-26), 14.2 (C-27), 101.0 (Glc C-1′), 76.9 (C-2′), 77.1 (C-3′), 79.6 (C-4′), 76.8 (C-5′), 60.3 (C-6′), 103.4 (Rha C-1′′), 76.2 (C-2′′), 73.9 (C-3′′), 75.0 (C-4′′), 70.0 (C-5′′), 17.3 (C-6′′), 104.0 (Rha C-1′′′), 73.5 (C-2′′′), 74.3 (C-3′′′), 75.3 (C-4′′′), 70.1 (C-5′′′), 17.2 (C-6′′′) 104.6 (Glc C-1′′′′), 76.2 (C-2′′′′), 79.0 (C-3′′′′), 73.1 (C-4′′′′), 79.5 (C-5′′′′), 61.1 (C-6′′′′).

Compound 9: white powder, HRMS *m/z* 1065.5480 [M + H] $^+$ (calcd for $C_{51}H_{84}O_{23}$, 1064.5403). ^{1}H-NMR (500 MHz, DMSO-*d*$_6$): *δ* 4.93 (1H, *br s*, Glc-1′′′), 5.08 (1H, *br s*, Fuc-1′), 5.15 (1H, *br s*, Xyl-1′′), 5.31 (1H, *d*, *J* = 5.5 Hz, Glc-1′′′′), 5.72 (1H, *d*, *J* = 4.0 Hz). ^{13}C-NMR (125 MHz, DMSO-*d*$_6$): *δ* 79.8 (C-1), 36.5 (C-2), 68.9 (C-3), 44.1 (C-4), 140.6 (C-5), 121.2 (C-6), 33.1 (C-7), 33.5 (C-8), 49.6 (C-9), 40.6 (C-10), 23.7 (C-11), 40.1 (C-12), 40.4 (C-13), 55.8 (C-14), 31.1 (C-15), 79.5 (C-16), 62.7 (C-17), 15.9 (C-18), 19.3 (C-19), 40.2 (C-20), 16.2 (C-21), 109.7 (C-22), 31.1 (C-23), 27.4 (C-24), 34.6 (C-25), 73.9 (C-26), 16.3 (C-27), 101.2 (Fuc C-1′), 77.1 (C-2′), 85.1 (C-3′), 73.6 (C-4′), 70.2 (C-5′), 17.1 (C-6′), 103.4 (Xyl C-1′′), 73.9 (C-2′′), 76.6 (C-3′′), 69.5 (C-4′′), 66.0 (C-5′′), 103.0 (Glc C-1′′′), 76.0 (C-2′′′), 76.9 (C-3′′′), 71.6 (C-4′′′), 76.8 (C-5′′′), 61.2 (C-6′′′), 102.5 (Glc C-1′′′′), 74.3 (C-2′′′′), 76.5 (C-3′′′′), 74.4 (C-4′′′′), 76.1 (C-5′′′′), 60.9 (C-6′′′′).

Compound 14: white powder, HRMS *m/z* 1247.5687 [M + Na]$^+$ (calcd for $C_{57}H_{92}O_{28}$, 1224.5775). ^{1}H-NMR (500 MHz, DMSO-*d*$_6$): *δ* 5.02 (1H, *br s*, Glc-1′), 5.05 (1H, *br s*, Xyl-1′′′′), 5.10 (1H, *br s*, H-16), 5.20 (1H, *d*, *J* = 2.4 Hz, H-12), 5.27 (1H, *br s*, Rha-1′′′), 5.44 (1H, *br s*, Api-1′′′′′), 5.55 (1H, *d*, *J* = 1.8 Hz, Ara-1′′). ^{13}C-NMR data were shown in Table S1.

Compound 15: white powder, HRMS *m/z* 1255.5944 [M + H]$^+$ (calcd for $C_{58}H_{94}O_{29}$, 1254.5881). ^{1}H-NMR (500 MHz, DMSO-*d*$_6$): *δ* 5.01 (1H, *d*, *J* = 4.2 Hz, Glc-1′), 5.02 (1H, *d*, *J* = 4.8 Hz, Xyl-1′′′′′), 5.13 (1H, *d*, *J* = 4.8 Hz, Glc-1′′), 5.16 (1H, *d*, *J* = 4.8 Hz, H-16), 5.26 (1H, *d*, *J* = 5.4 Hz, H-12), 5.47 (1H, *br s*, Rha-1′′′′), 5.50 (1H, *d*, *J* = 1.8 Hz, Ara-1′′′). ^{13}C-NMR data were shown in Table S1.

Compound **16**: white powder, HRMS *m/z* 1409.6207 [M + Na] $^+$ (calcd for $C_{63}H_{102}O_{33}$, 1386.6303). ^{1}H-NMR (500 MHz, DMSO-*d*$_6$): *δ* 5.01 (1H, *d*, *J* = 4.2 Hz, Glc-1′), 5.07 (1H, *d*, *J* = 6.0 Hz, Xyl-1′′′′′), 5.12 (1H, *d*, *J* = 4.8Hz, Glc-1′′), 5.16 (1H, *d*, *J* = 5.4 Hz, H-16), 5.18 (1H, *d*, *J* = 2.4 Hz, H-12), 5.27 (1H, *br s*, Rha-1′′′′), 5.41 (1H, *d*, *J* = 3.6 Hz, Api-1′′′′′′), 5.52 (1H, *d*, *J* = 1.8 Hz, Ara-1′′′). ^{13}C-NMR data were shown in Table S1.

Compound **17**: white powder, HRMS *m/z* 1393.6238 [M + Na] $^+$ (calcd for $C_{63}H_{102}O_{32}$, 1370.6354). ^{1}H-NMR (500 MHz, DMSO-*d*$_6$): *δ* 5.02 (1H, *br s*, Glc-1′), 5.02 (1H, *br s*, Xyl-1′′′′′), 5.03 (1H, *d*, *J* = 4.8 Hz, Glc-1′′), 5.07 (1H, *d*, *J* = 6.0 Hz, H-16), 5.17 (1H, *d*, *J* = 2.4 Hz, H-12), 5.25 (1H, *br s*, Rha-1′′′′), 5.43 (1H, *d*, *J* = 3.6 Hz, Api-1′′′′′′), 5.53 (1H, *br s*, Ara-1′′′). ^{13}C-NMR data were shown in Table S1.

Compound 18: white powder, HRMS *m/z* 1387.6377 [M + H] $^+$ (calcd for $C_{63}H_{102}O_{33}$, 1386.6303). ^{1}H-NMR (500 MHz, DMSO-d_6): δ 5.02 (1H, *d*, *J* = 2.4 Hz, Glc-1′), 5.03 (1H, *d*, *J* = 8.1 Hz, Xyl-1′′′′′), 5.06 (1H, *d*, *J* = 3.6 Hz, Glc-1′′), 5.07 (1H, *d*, *J* = 5.4 Hz, H-16), 5.18 (1H, *d*, *J* = 1.8 Hz, H-12), 5.25 (1H, *br s*, Rha-1′′′′), 5.42 (1H, *d*, *J* = 4.2 Hz, Api-1′′′′′′), 5.53 (1H, *d*, *J* = 1.8 Hz). ^{13}C-NMR data were shown in Table S1.

Compound 20: light yellow powder, HRMS *m/z* 433.1131 [M + H] $^+$ (calcd for $C_{21}H_{20}O_{10}$, 432.1056). ^{1}H-NMR (500 MHz, DMSO-d_6): δ 6.50 (1H, *s*, H-3), 6.77 (1H, *s*, H-8), 6.93 (2H, *d*, *J* = 7.5 Hz, H-3′, H-5′), 7.93 (2H, *d*, *J* = 7.5 Hz, H-2′, H-6′), 13.56 (1H, *s*, 5-OH). ^{13}C-NMR (125 MHz, DMSO-d_6): δ 163.4 (C-2), 102.7 (C-3), 181.9 (C-4), 160.7 (C-5), 109.0 (C-6), 163.4 (C-7), 93.7 (C-8), 103.2 (C-4a), 156.3 (C-8a), 121.1 (C-1′), 128.1 (C-2′), 116.0 (C-3′), 161.3 (C-4′), 116.0 (C-5′), 128.5 (C-6′), 73.1 (Glc C-1′′), 70.6 (C-2′′), 79.0 (C-3′′), 70.2 (C-4′′), 81.6 (C-5′′), 61.5 (C-6′′).

Compound 21: light yellow powder, HRMS *m/z* 433.1128 [M + H] $^+$ (calcd for $C_{21}H_{20}O_{10}$, 432.1056). ^{1}H-NMR (500 MHz, DMSO-d_6): δ 6.25 (1H, *s*, H-6), 6.76 (1H, *s*, H-3), 6.90 (2H, *d*, *J* = 7.0 Hz, H-3′, H-5′), 8.02 (2H, *d*, *J* = 5.5 Hz, H-2′, H-6′), 13.16 (1H, *s*, 5-OH). ^{13}C-NMR (125 MHz, DMSO-d_6): δ 163.8 (C-2), 102.3 (C-3), 182.0 (C-4), 160.4 (C-5), 98.4 (C-6), 161.3 (C-7), 104.7 (C-8), 104.7 (C-4a), 156.0 (C-8a), 121.6 (C-1′), 128.9 (C-2′), 115.9 (C-3′), 161.3 (C-4′), 115.9 (C-5′), 128.9 (C-6′), 73.4 (Glc C-1′′), 70.9 (C-2′′), 78.7 (C-3′′), 70.5 (C-4′′), 81.8 (C-5′′), 61.3 (C-6′′).

Compound 22: white powder, HRMS *m/z* 457.1658 [M-H] $^-$ (calcd for $C_{21}H_{30}O_{11}$, 458.1788). ^{1}H-NMR (500 MHz, DMSO-d_6): δ 5.16 (1H, *s*, H-9a), 5.20 (1H, *d*, *J* = 4.0 Hz, H-9b), 5.91 (1H, *m*, H-8), 6.56 (1H, *dd*, *J* = 2.0, 1.5 Hz, H-6), 6.63 (1H, *d*, *J* = 2.0 Hz, H-5), 7.02 (1H, *d*, *J* = 7.0 Hz, H-2). ^{13}C-NMR (125 MHz, DMSO-d_6): δ 135.1 (C-1), 117.4 (C-2), 144.2 (C-3), 147.2 (C-4), 116.5 (C-5), 119.7 (C-6), 39.4 (C-7), 138.4 (C-8), 116.0 (C-9), 103.2 (Glc C-1′), 72.5 (C-2′), 76.2 (C-3′), 70.5 (C-4′), 76.1 (C-5′), 67.2 (C-6′), 101.2 (Rha C-1′′), 71.0 (C-2′′), 71.2 (C-3′′), 73.8 (C-4′′), 68.9 (C-5′′), 18.4 (C-6′′).

Compound 24: white powder, HRMS *m/z* 203.0824 [M-H] $^-$ (calcd for $C_{11}H_{12}N_2O_2$, 204.0899). ^{1}H-NMR (500 MHz, DMSO-d_6): δ 7.01 (1H, *m*, H-6), 7.09 (1H, *m*, H-7), 7.23 (1H, *d*, *J* = 2.0 Hz, H-4), 7.38 (1H, *d*, *J* = 8.0 Hz, H-8), 7.57 (1H, *d*, *J* = 7.6 Hz, H-5), 8.23(1H, *s*, NH), 11.08 (1H, *s*, COOH). ^{13}C-NMR (125 MHz, DMSO-d_6): δ 170.9 (C-1), 136.3 (C-2), 127.1 (C-3), 125.0 (C-4), 121.2 (C-5), 118.7 (C-6), 118.3 (C-7), 111.6 (C-8), 106.7 (C-9), 52.6 (C-10), 26.2 (C-11).

Compound 25: white powder, HRMS *m/z* 268.1048 [M + H] $^+$ (calcd for $C_{10}H_{13}N_5O_2$, 267.0968). ^{1}H-NMR (500 MHz, DMSO-d_6): δ 8.34 (1H, *s*, H-8), 8.13 (1H, *s*, H-2), 5.87 (1H, *d*, *J* = 5.0 Hz, H-1′), 4.60 (1H, *br s*, H-2′), 4.14 (1H, *br s*, H-3′), 3.97 (1H, *m*, H-4′), 3.66 (1H, *m*, H-5′), 3.55 (1H, *m*, H-5′′). ^{13}C-NMR (125 MHz, DMSO-d_6): δ 152.4 (C-2), 149.1 (C-4), 119.4 (C-5), 156.2 (C-6), 140.0 (C-8), 87.9 (C-1′), 73.5 (C-2′), 70.7 (C-3′), 86.0 (C-4′), 61.7 (C-5′).

Compound 26: colorless powder, HRMS *m/z* 243.0857 [M + Na] $^+$ (calcd for $C_9H_{16}O_6$, 220.0947). ^{1}H-NMR (500 MHz, DMSO-d_6): δ 4.92 (1H, *d*, *J* = 8.5 Hz, Glc-1′), 5.84 (1H, *br s*, H-1a), 6.76 (1H, *d*, *J* = 7.5 Hz, H-1b), 7.53 (1H, *d*, *J* = 7.0 Hz, H-2). ^{13}C-NMR (125 MHz, DMSO-d_6): δ 114.5 (C-1), 131.5 (C-2), 69.6 (C-3), 103.8 (Glc C-1′), 72.4 (C-2′), 78.5 (C-3′), 69.8 (C-4′), 81.1 (C-5′), 60.5 (C-6′).

Table S1. ^{13}C-NMR data of compounds 14–18.

Position	14	15	16	17	18	Position	14	15	16	17	18
1	44.3	44.1	44.1	43.2	44.2	16	73.7	73.5	73.5	73.7	73.7
2	68	68.1	68	68.7	68	17	48.1	48.2	48.2	48.1	48.1
3	81.3	81.3	81.3	81.3	81.3	18	40.21	40.38	40.37	40.13	40.2
4	46.7	46.9	46.9	41.6	46.7	19	46.1	46.1	46.1	46	46.2
5	46.4	46.4	46.4	46.3	46.2	20	30.3	30.3	30.3	30.3	30.3
6	18.1	18.1	18.1	17.6	18	21	35	35	35	35	35

7	32.9	32.9	32.9	32.9	32.9	22	31.1	31.1	31.1	31.1	31.1
8	40.1	40.1	40.1	40.1	40.1	23	61.5	61.4	61.2	67.2	61.1
9	46.52	46.4	46.9	46.2	46.6	24	67.2	67.2	67.2	14.5	67.2
10	36.3	36.7	36.7	35.9	36.2	25	17.6	17.6	17.6	16.7	18
11	23.1	23.1	23.1	23.1	23.1	26	16.6	16.7	16.7	16.7	16.6
12	122	122	122	122	122	27	26.4	26.4	26.4	26.5	26.4
13	143.5	143.6	143.5	143.4	143.5	28	174.7	174.7	174.7	174.7	174.7
14	41.2	41.3	41.3	41.2	41.2	29	32.6	32.6	32.6	32.2	32.6
15	34.7	34.7	34.7	34.7	34.7	30	24.2	24.2	24.2	24.3	24.2
Glc-1 (terminal)	104.3	104.1	103.2	103.6	104.2	Ara-1	91.7	91.8	91.8	91.7	91.7
2	73.8	73.3	73.8	73.9	73.8	2	74.2	74.3	74.3	74.2	74.2
3	76.8	76.9	76.1	76	76	3	70.2	69.2	69.9	70	70.2
4	70.5	70.6	70.6	69	70.5	4	64.2	64.5	64.3	64.2	64.2
5	75.9	74.3	74.9	75.1	76	5	61.1	61.3	61	61	60.9
6	61	61	61.1	61.1	88.4	Rha-1	99.4	99.5	99.5	99.3	99.4
Glc-1 (terminal)		103.1	103.1	103.2	103.5	2	70.2	70.1	70.5	70.5	70.2
2		73.8	73.6	73.7	73.7	3	72.4	72.3	72.3	72.4	72.4
3		74.9	76	76.8	76.4	4	79.1	76.6	79.1	79.1	79.1
4		69.9	70.3	70.2	70.3	5	67.7	67.1	67.9	67.6	67.7
5		76.6	76.6	76.5	76.9	6	17.3	17.6	17.6	17.6	17.6
6		61.1	61.1	61.1	61						
Xyl-1	104.8	105.1	104.8	104.8	104.8	Api-1	108.9	108.9	108.9	108.9	108.9
2	72.2	73.9	73.8	73.7	73.8	2	76	76	76	76	76
3	80.8	76	80.9	80.9	80.8	3	76.9	76.9	76.9	76.8	76. 9
4	68	69.5	69.2	68	68.6	4	73.9	73.9	73.9	73.7	73.9
5	65.8	66	65.8	65.8	65.8	5	63.8	63.8	63.8	63.8	63.8
